# Policy-making and implementation for newborn bloodspot screening in Europe: a comparison between EURORDIS principles and UK practice

**DOI:** 10.1016/j.lanepe.2023.100714

**Published:** 2023-08-10

**Authors:** Silvia Lombardo, Farah Seedat, David Elliman, John Marshall

**Affiliations:** a UK National Screening Committee, Department of Health and Social Care, 39 Victoria Street, London SW1H 0EU, UK; b St. George’s, University of London, Cranmer Terrace, London SW17 0RE, UK

**Keywords:** Newborn screening, UK National Screening Committee, EURORDIS, Rare disease

## Abstract

Newborn bloodspot screening (NBS) policy is a contentious area in Europe. Variation in the screening panels on offer, in the approach to evidence assessment and in the use of health economic modelling are some of the issues which are debated on the topic. In this paper we focus on a set of patient-driven principles for newborn screening published by EURORDIS and use these as a reference point for exploration and comparison with NBS policy development and screening practice in the UK. In doing so, we share UK practice; we note the UK is generally well aligned with many of the recommended principles, but we also discuss areas of controversy and challenges. Some of these, like ‘actionability’, will undoubtedly continue to be debated and may never reach consensus. For others, such as patient and public voice participation in newborn screening systems, there are opportunities to continue improving existing processes and developing new mechanisms for stakeholder participation. Screening bodies in other European countries should also compare their policy-making and implementation practices with the EURORDIS principles to stimulate further discussion on the challenges and opportunities of newborn screening and provide a cross-European baseline.

## Introduction

Collectively, rare and ultra-rare conditions have a substantial impact on society. Babies affected by these conditions, their families and caregivers face challenges of often long diagnostic delays, poor care management and a lack of effective treatments. Although definitions differ, it has been proposed that rare conditions affect less than 1 in 2000 people in Europe and ultra-rare conditions affect less than 1 in 50,000 people.^1^, ^2^, ^3^, ^4^ They include metabolic, neurological and neuromuscular conditions, often affecting multiple organs and body systems. Despite global efforts to improve diagnostic capabilities and develop new therapies, affected families continue to face unmet clinical and social needs, which place a considerable psychological and economic burden on them.^5^ Patient groups, industry representatives and clinicians increasingly target newborn bloodspot screening (NBS) as a solution.^6^^,^^7^ NBS seeks to identify babies with rare and ultra-rare conditions soon after birth through abnormalities in their blood to enable early intervention. For some conditions, this can consequently improve health outcomes and prevent severe disability or premature death. However, an ill thought-out programme may do more harm than good and may be ethically challenging to some stakeholder groups. A recent summary of screening for three lysosomal storage disorders draws attention to the potential for NBS to generate limited health benefits for screen positive cases set against false positive test results, overdiagnosis, uncertain prognoses, and uncertain options for treatment and its timing. This can result in a burden on the individual, the family and the health system.^8^

In line with other screening programmes, to decide which conditions to include in NBS, most decision-making and advisory bodies have adapted the Wilson and Jungner (W&J) principles.^9^ These longstanding criteria cover considerations in relation to the epidemiology and natural history of a condition, the suitability and acceptability of tests, the availability of an accepted treatment, and the cost-effectiveness of the entire programme. In the UK for example, the UK National Screening Committee (UKNSC) has adapted the W&J principles into 20 criteria. These are used to evaluate the viability of population screening programmes and to advise ministers and the National Health Service (NHS) in the four UK countries (See Supplementary Table S1). Similar to the UK, in Spain, the principles have been adapted into 18 criteria plus an additional seven implementation requirements, expanding W&J’s original scope.^10^

Despite this broad consistency, there is international variation in NBS policy and practice. Recent efforts to bring consistency to NBS have included the development of sets of principles for decision-making and implementation. An important example of this was published by EURORDIS, a patient-driven alliance of 1000 rare disease patient organisations from several countries whose aim is to improve the lives of people living with rare diseases in Europe.^11^ In 2021, EURORDIS produced 11 key principles for NBS, recommended for adoption throughout Europe (See Supplementary Table S2). The principles promote consistency in NBS across a broad range of issues including governance arrangements, the screening panel and the timing of the test.^12^ More recently, Scarpa et al. (2022)^13^ have also put forward some guiding principles (See Supplementary Table S3). They emphasised the need for transparent and robust processes for selecting new conditions, consistent case definitions for conditions included in the panel, the importance of sharing information with parents, and clear policies to store and access residual bloodspot samples.

In this paper, we focus on the EURORDIS principles as a comprehensive reference point to explore how NBS policy development and screening practice in the UK compares with a set of principles led by patients. In doing so, we share screening practices from the UK and discuss areas of controversy and challenges.

## Search strategy and selection criteria

References for this personal view were identified through searches of the authors’ own files and from reference lists supporting evidence reviews commissioned by the UKNSC. Papers published were not restricted to the English language. The final reference list was generated on the basis of relevance to the scope of this manuscript.

## EURORDIS principles for newborn bloodspot screening

EURORDIS calls for a harmonised and uniform approach to NBS across Europe, with a view to reducing variations between the policies and implementation of newborn screening programmes. Their principles focus on best practice for screening policy-making and implementation at national level^12^ (See Supplementary Table S2).

The policy-making principles focus attention on the type of conditions that NBS should identify. These are “actionable” conditions. Actionable in this context means (a) conditions where early interventions lead to health gains for the newborn, (b) conditions where early diagnosis avoids the lengthy diagnostic delay and (c) conditions where parents will have reproductive options during subsequent pregnancies (principle 1). The importance of basing NBS on the best available evidence, including health economic evidence is addressed in two principles (principles 5 and 7). The need for evaluations to be undertaken within an independent and impartial process is considered to be an important feature of NBS systems (principle 2). Finally, there is an emphasis on stakeholder participation in the NBS evaluation and implementation processes with families of healthy newborns and those who receive a false positive screening result included at all stages (principle 4). This is because all will require follow-up and support under a comprehensive screening programme.

In terms of screening practice, EURORDIS advocates that all European nations should have standards addressing the timing, sample collection methods, follow-up, and information shared with parents (principle 9). It is not clear whether they should be identical in content or just cover the same areas. They also emphasise the need to provide families of newborns diagnosed through NBS with psychological, social and economic support, as well as provision of information and education on rare diseases and the whole screening process to all stakeholders (principles 3 and 8). EURORDIS also identifies the need to embed robust and transparent governance processes across the NBS pathway, with clearly defined roles, responsibilities, accountability and communication networks (principle 6). Other aspects include the importance of using residual bloodspot samples for research purposes and the use of registries to gather epidemiological data and treatment outcomes to generate evidence relating to NBS (principles 10 and 11).^12^

## The landscape of newborn bloodspot screening in the UK and its alignment with EURORDIS

In terms of policy-making governance (principle 6) in the UK, the UKNSC is responsible for making recommendations on whether or not to screen for an NBS condition. The Committee is an independent scientific advisory committee^14^ sponsored by the Department of Health and Social Care, and is accountable to the Chief Medical Officers in each of the four UK nations. The health department in each UK nation is responsible for setting its screening policy with the agreement of their respective ministers, considering advice from UKNSC.^15^ The NHS in each nation then has the responsibility to implement the screening policies, promote equal access to screening and provide high quality information to enable informed choice.

In the UK, all elements of the screening and subsequent management pathway are free at the point of delivery and provided by the NHS, aligning with EURORDIS principle 3. This does not only cover medical interventions, but includes other support from public bodies such as psychological, social and educational. In keeping with EURORDIS principles 2, 6 and 9, in the UK responsibilities are decided within each of the four nations, as are the screening standards covering all aspects of the screening pathway (coverage, test, referral, intervention or treatment) and how they should be monitored.^16^, ^17^, ^18^ Furthermore, in line with EURORDIS principle 8, whenever new programmes have been planned in the UK, all stakeholders have been involved via an iterative process in the planning of the implementation. This includes information and education materials about the conditions being screened for, the test and the pathway. All information and education materials have been designed and field tested with the relevant stakeholders and modified based on their feedback. This is considered essential for efficient implementation of the programme and to enable parents to make informed decisions about NBS.

In keeping with EURORDIS principles 5 and 7, the UKNSC is responsible for reviewing the scientific literature in relation to key screening criteria (See Supplementary Table S1) in order to assess the possible introduction, modification and cessation of national population screening programmes. These criteria cover the condition, the test, the treatment and the effectiveness of screening programmes in the UK, and aim to ensure that their benefits outweigh the harms at a reasonable cost.

Finally, the UKNSC policy-making processes are relevant to EURORDIS’ aspiration that all stakeholders should be included in the different stages of the NBS process (principle 4). The Committee hosts an annual call for topics where anyone can suggest a new screening programme, propose an early update to an existing topic or a modification to a current screening programme. The UKNSC also has a commitment to review existing recommendations on a regular basis. As part of the review process, the UKNSC hosts a 3-month public consultation for every screening recommendation.^19^ Any member of the public can share their views on the standard of the evidence review and the review’s conclusions, highlight any omitted evidence, and provide expert opinion, clinical experience and patient and families accounts. The review, along with the stakeholder comments, is then presented to UKNSC members for discussion and decision-making.^19^ From topic selection to completion of evidence review, the fetal, maternal and child health group advises the UKNSC on all matters relating to NBS. This group includes a broad range of clinical, user, academic, economic and ethical expertise to provide input and scrutinise policy development.^20^ If an evidence review identifies a promising candidate for screening, the UKNSC may commission further in-depth analyses. These may include additional evidence reviews, cost-effectiveness assessments, decision analytic modelling or in-service evaluations. These projects provide forums for more detailed and longer-term input with a wider range of stakeholders.

UKNSC has initiated further stakeholder involvement by setting up a bloodspot task group to provide a managed forum for discussion of practical and innovative approaches to facilitate research and evidence development in rare diseases. The group brings together a wealth of expertise in newborn screening, including patient and public voice (PPV) representatives, paediatricians, academics (including researchers, methodologists, and health economists), ethicists, quality assurance and laboratory professionals and geneticists.^21^

## Challenges for newborn bloodspot screening in the UK and Europe

Here we consider some areas of divergence between the principles and UK policy-making and practice. A comparison between the EURORDIS principles and the UK approach is summarised in Table 1.

**Table 1:** Comparison between EURORDIS principles and the UK approach.

EURORDIS	UK	Alignment^a^
Key Principle 1: Screening should identify opportunities to help the newborn and the family as broadly as possible. That is, screening should identify actionable diseases including treatable diseases	The UKNSC criteria agree that newborn screening which directly improves the wellbeing of the child should be considered ‘actionable’. Evidence relating to wider benefits of screening, for example those relating to family members, are taken into account where available. However, in line with the UKNSC’s first ethical principle and professional consensus in the UK, the UKNSC criterion 9 is clear that without direct benefit to the person being screened—in this case the child—a screening programme is not acceptable. If the intervention does not need to be initiated until the person is able to make an informed choice, professional guidance states that testing should be delayed until the individual themself is capable of making that choice	Partial
Key Principle 2: NBS should be organised as a system with clearly defined roles, responsibilities, accountability and communication pathways that are embedded into the national health care system and recognised as a mechanism for earlier diagnosis of actionable conditions as part of the broader care pathway	In the UK, NBS is an intensively quality assured process which includes a full end to end pathway. This pathway is managed from the invitation to take part in screening (which is offered to every parent), testing, further testing as required, referral, diagnosis and treatment by appropriate newborn specialist clinicians. Roles and responsibilities are clearly defined, decided within each of the four nations and embedded into the NHS (see also entry below in relation to EURORDIS key principle 6) It is important to note that there is full alignment in relation to the key principle; the implementation of the principle is a separate point, and it might not be consistently executed across the whole NHS	Full
Key Principle 3: The family of the newborn who has been diagnosed through NBS should be provided with psychological, social and economic support by the competent national health authorities	In the UK, all elements of the screening and subsequent management pathway are free at the point of delivery and provided by the NHS. This does not only cover medical interventions but includes other support from public bodies such as psychological, social and educational It is important to note that there is full alignment in relation to the key principle; the implementation of the principle is a separate point, and it might not be consistently executed across the whole NHS	Full
Key Principle 4: All stakeholders should be included in the different stages of the NBS process	In the UK, mechanisms are in place to include stakeholders at all points of policy making and programme implementation. The approach to stakeholder involvement aims to be proportionate, flexible, responsive and transparent, as well as inclusive, engaging with a range of organisations, communities and voices (see UKNSC stakeholder engagement strategy). The UKNSC’s engagement processes include consultative and participatory mechanisms. However some specific EURORDIS recommendations have not been uniformly integrated into UK practice. For example, this includes the recommendation that there ‘should be a minimum representation of patient associations and professional experts specifically for the conditions to be discussed, included on committees responsible for the evaluation of NBS programme’.	Partial
Key Principle 5: Transparent and robust governance for expanding NBS programmes is needed. Every country/region should have a clearly defined transparent, independent, impartial and evidence-based process for deciding which conditions are covered by the NBS programme that includes all stakeholders	The UKNSC follows a clearly defined and transparent evidence review process to assess the available evidence (national and international) for screening using the UK NSC criteria for appraising the viability, effectiveness and appropriateness of a screening programme. The main divergence relates to the inclusion of stakeholders which is addressed at principle 4.	Partial
Key Principle 6: Governance of NBS programmes should be explicit, comprehensive, transparent and accountable to national authorities	The UKNSC, an independent Scientific Advisory Committee sponsored by the four UK Departments of Health, is responsible for making recommendations on whether or not to screen for an NBS condition and is accountable to the CMOs in each of the four UK nations. The health department in each UK nation is responsible for setting its screening policy with the agreement of their respective ministers, considering advice from UKNSC. The NHS in each nation then has the responsibility to implement the screening policies, promote equal access to screening, provide high quality information to enable informed choice	Full
Key Principle 7: The evaluation process on the inclusion/exclusion of diseases in NBS programmes needs to be based on the best available evidence, reflecting health economic evidence but not determined only by health economics	In the UK, decisions on NBS are based on the best available evidence, including health economic evidence and a comparison of the impact of early and late diagnosis. The use of modelling to estimate the effects of screening and inform decision-making is increasingly moving to the centre of UKNSC practice on rare diseases, given how these conditions present significant challenges in evidence-based medicine because of the large sample sizes required to produce meaningful results in research studies. However, the UKNSC would not recommend screening if this could not be justified from a cost-effectiveness perspective. Also, there may be divergence between EURORDIS and UKNSC on what constitutes best available evidence. The UKNSC would not recommend a screening programme if the best available evidence does not provide sufficient reassurance that it would do more good than harm.	Partial
Key Principle 8: Information and education of all stakeholders on rare diseases and the whole NBS process is essential for a broad and fair implementation of NBS programmes	Information and education materials about the conditions being screened for, the test and the pathway are designed and also field tested with the relevant stakeholders and modified based on their feedback. This is considered essential for efficient implementation of the programme and to enable parents to make informed decisions about NBS	Full
Key Principle 9: European-wide standards addressing the timing, sample collection methods, follow-up, and information shared with parents are needed to guarantee uniformity and quality throughout the process	EURORDIS advocates for each EU Member State to provide standards to guarantee a certain level of consistency in the quality and implementation of the different steps and procedures involved in NBS. This aligns with UK practice with screening standards covering all aspects of the screening pathway, how they should be monitored, and quality assured being decided within each of the four nations. The UK standards relate to: coverage, test, referral, intervention or treatment. Each year, an annual report is published describing the timeliness of various milestones on the screening pathway, including receipt of results in the laboratory, results becoming available and when the baby and family are seen by the appropriate specialist team. In line with other UK countries, in England, there is a Newborn Blood Spot Failsafe System, an IT solution that reduces the risk of babies missing or having delayed NBS. It supports the maternity service responsibility for making sure NBS is offered and that samples are taken and received in the laboratory on time. It is important to note that there is full alignment in relation to the part of the key principle which relates to the standards. However, EURORDIS expands this principle further and recommends a registry to “be created in order to have systematic follow-up of all newborns detected with a condition through NBS”. Because this is not part of the current practice in the UK, the overall alignment is partial	Partial
Key Principle 10: Blood spot samples should be stored in national biobanks for quality control and research purposes while ensuring appropriate measures for data access as well as robust safeguards for data protection and privacy are in place	Currently in the UK, DBS are stored for at least five years, apart from Scotland where they are stored indefinitely. There are currently some limitations in DBS storage in England. DBS are not stored under uniform conditions in a centralised location, which might in part hinder the reliable use of DBS for research	Partial
Key Principle 11: ERN affiliated centres should be integrated in the care pathways of the different Healthcare systems and should be considered as preferential partners in providing recommendations on NBS policies	There is interaction between ERNs and UK experts. However, there are no ERN affiliated centres in the UK and preferential partnership with a single sector grouping has not been integrated into the UKNSC decision-making processes	Not applicable

Abbreviations: CMOs, Chief Medical Officers; DBS, dried bloodspots; ERN, European Reference Network; EU, European Union; NBS, newborn bloodspot screening; NHS, National Health Service; UKNSC, UK National Screening Committee.

a **Full alignment**: the UK approach and the EURORDIS principles are in agreement. **Partial alignment**: the UK approach and the EURORDIS principles broadly align but they may differ in some respects. **Not applicable:** the UK is out of the EU and there are no ERN affiliated centres in the UK.

### What does “actionable” mean?

EURORDIS and the UKNSC agree that newborn screening which directly improves the wellbeing of the child, whether that be by drugs, gene therapy, diet or other intervention, should be considered ‘actionable’.

EURORDIS also proposes that benefit to the family per se is sufficient justification for screening as even without a cure or a treatment, early diagnosis may enable families to avoid a long diagnostic delay and make informed reproductive choices for subsequent pregnancies.

On the other hand, the UKNSC’s first ethical principle and professional consensus in the UK is clear that without direct benefit to the person being screened, a screening programme is not acceptable.^22^ Wider benefits to the family can strengthen the case for NBS but, alone, cannot provide justification for recommending screening. If the intervention does not need to be initiated until the person is able to make an informed choice, professional guidance states that testing should be delayed until this point.^23^^,^^24^ Retention of the focus on a direct benefit for the screened individual is grounded in the distinctive nature of screening in which the invitation to test is initiated by the health service in populations without a prior indication or symptom.^25^ The UKNSC and EURORDIS therefore diverge significantly on the issue of ‘actionability’. The crux of the divergence is that the EURORDIS principles broaden the concept of who should be the recipient of benefit to encompass the family unit and separate this from the screened baby (principle 1).

A diversity of views on this topic has been noted in the wider literature^26^, ^27^, ^28^ and this may reflect a more deep-seated divergence on ‘whose view counts’ when considering the acceptable outcomes of a screening programme. In this respect, the EURORDIS principles prioritise the perspective of the rare diseases community, in particular those who have been directly affected by the condition. This contrasts with the UKNSC criteria and ethical framework which emphasise the need to include the perspectives of a broader group of stakeholders, such as the public health community, and the whole population offered screening. Indeed, the UKNSC position on actionability reflects an attempt to balance an established perspective on screening, which is often associated with public health principles,^8^^,^^29^^,^^30^ with that of the rare disease community.

A recent public dialogue exercise in the UK offers some insight into this. Genomics England Ltd (GEL) is in the process of co-designing and running a research study to explore the benefits, challenges, and practicalities of sequencing and analysing the genomes of newborns.^31^ The public dialogue was a joint exercise sponsored by GEL and the UKNSC as part of this research. Views were sought from members of the general public, parents of children with genetic disorders and some individuals with such conditions.^32^ While recognising the potential for whole genome sequencing in neonates to bring benefits to other members of the family, the majority of those taking part considered that all the included conditions should have an impact in early childhood and there should be effective management available.

The dialogue underlined the close connection between concepts of actionability and the acceptability of screening programmes, including NBS. It also drew attention to the diverging perspectives on these issues. Further study of this complicated area has been proposed^26^ and, given its importance, this would seem an appropriate response before considering whether alignment with this EURORDIS principle is the correct course.

### Consistency in screening panels

EURORDIS promotes consistency in the screening panel adopted in European nations. However, across Europe the number of conditions included in recommended panels varies from one to over 30.^33^^,^^34^ Factors affecting this include disease incidence, the relative importance attached to sensitivity and specificity, the level of evidence required and the assessment of cost-effectiveness. Varying conceptions of actionability may also be a factor in this. Other reasons may relate to variations in reporting of screening activities and the type of recommendations which are made. For example, when comparing the number of conditions included in national screening panels, a careful distinction should be made between screening pilots, in-service evaluations and fully-established screening programmes. Attention to the intra-jurisdiction geographies of such efforts is also important. Published reports appear to have become more sensitive to this issue as a comparison between two surveys of practice highlights.^33^^,^^35^ The later survey reported that, in Spain, the national recommendation and implementation of NBS includes seven conditions with an additional 26 conditions being piloted or being offered at regional level.^33^ This compares to the earlier report in which this distinction was not made.^36^ Furthermore, there may be variation in the level at which screening services are implemented in different countries. In the UK, NBS is implemented at the national level in each of the four nations with reference to the panel recommended by the UKNSC and provided that a national end-to-end screening pathway can be established. By contrast, in some European nations, recommendations are made centrally but adoption of the panel is devolved to lower (regional or municipal) levels. This can lead to variation in NBS screening panels within countries in terms of implementation timescales and selection of screening panels.^37^^,^^38^ Finally, incidental findings from screening are not considered to be a part of the screening panel in the UK, but it is unclear whether this is the case in reports of screening panels elsewhere. This is important as the sensitivity of screening, when a condition is an incidental finding, may be lower as in the case of galactosaemia and tyrosinaemia as incidental products of phenylketonuria screening. Therefore, the extent to which consistency in NBS panels is desirable and whether variation should be expected and justified remains a challenge, one that would benefit from greater understanding of the reasons for variations in policy-making and practice across countries.

### Evidence assessment

EURORDIS and the UKNSC both emphasise that decisions on NBS need to be based on the best available evidence, including health economic evidence and a comparison of the impact of early and late diagnosis. In this respect, significant variation across policy-making bodies was reported in a recent systematic review. This found that 42% of recommendations by national policy-making bodies did not take account of evidence on test accuracy, 36% did not review evidence about whether early treatment improves health outcomes, and 76% did not consider evidence about potential harms of overdiagnosis.^39^

Such differences in the criteria which are assessed in decision-making, the methods of assessment and the processes in which decision-making takes place can lead to different numbers of conditions being included in NBS panels. The UK is often considered an outlier by screening for fewer conditions compared to other European countries. This may be because the full range of assessment criteria are considered and valued in the UKNSC’s decision-making processes. Interestingly, the EURORDIS principles do not mention the word ‘harm’. False positive results, i.e. where a condition is suspected on screening, but not confirmed on diagnostic testing, are mentioned in this context, but issues such as overdiagnosis, incidental findings, clinically uncertain outcomes of screening and diagnostic tests, and impact on the wider health system are not considered. This is in keeping with the results of the systematic review,^39^ and may reflect a broader trend towards an emphasis on benefits in published discussions of early detection strategies.^7^

In terms of methodology, as evidence synthesis methods and processes evolve, there is debate as to what constitutes an optimum approach to assessment. A case study in the UK, comparing systematic and rapid review methodologies used to review the evidence on the use of succinylacetone-based screening tests for tyrosinaemia type 1^40^ suggested that systematic review methods captured the nuances of the evidence base most comprehensively. However, rapid reviews may provide policy-making bodies with sufficient information at certain points in the decision-making process. In the UK, they are used to filter a large number of topics with the intention of prioritising those with positive outcomes and more substantial evidence bases for further exploration. In addition, the issue of sufficiency of robust evidence has been highlighted as a problem in NBS decision-making. In the discussion on the rapid expansion of the US NBS programme, the tendency to rely on expert opinion over scientific research was noted as a particular concern.^29^ This tendency has found a resonance in the UK debate on NBS policy-making and is something which the UKNSC continues to struggle with, given the commitment to maintain high evidential standards for screening programmes. This commitment is made, in large part, because screening programmes invite ostensibly healthy populations for testing but also because of the challenges of programme cessation where they are found to be doing more harm than good.^41^, ^42^, ^43^

Moreover, evidence assessment methods and decision-making processes used in different countries often remain unpublished. The discussion about variation in NBS evidence assessment would therefore benefit from decision-making bodies sharing information about the range of viewpoints included (e.g. population health), criteria used, the type of review methodologies employed, the level of evidence required for recommendations, mechanisms for incorporating patient experience and professional opinion, how uncertainty in the evidence is handled to ensure that recommendations are robust, and how outcomes are monitored and reported over time.

### Modelling studies in decision-making

Rare and ultra-rare diseases present significant challenges in evidence-based medicine because of the large sample sizes and long timescales required to produce meaningful results. This in turn limits the quality of information available to inform estimates of the impact of screening. This problem has been noted for many years^29^^,^^44^ and the omission of key issues such as test accuracy and harms of screening in decision-making may simply reflect an accommodation to it. Nevertheless, the difficulty of generating high quality evidence does not remove the obligation on decision-makers to explain the aims and rationale of screening programmes and to quantify their effects.^45^ It is therefore interesting that the EURORDIS principles include the use of modelling to estimate the effects of screening and inform decision-making. This approach is increasingly moving to the centre of UKNSC practice on rare diseases.^46^ This is because modelling studies use a set of techniques and methods to synthesise evidence from different sources with expert opinion, enabling the simulation of comparisons which are unachievable using primary research methods. This approach has been used in UKNSC evaluations of NBS for SCID and, most recently, tyrosinaemia type 1. Modelling is strongly associated with evaluation of the cost-effectiveness of healthcare interventions. This is an important element of NBS decision-making in the UK and the UKNSC would not recommend screening if this could not be justified from a cost-effectiveness perspective. This is because the inappropriate diversion of resources from the provision of other, perhaps more effective, health interventions is a potential harm of screening.

However, the use of modelling can extend beyond economic analyses and modelling studies have proved to be a useful tool, helping to develop a set of baseline expectations about what screening may achieve compared to current practice and provided a way in which expertise from clinical, academic and patient stakeholders can be incorporated into policy recommendations. However, a number of challenges have been identified with this approach in the context of NBS. In particular, a consensus on what constitutes a ‘good’ model does not appear to have been established. For example, a recent review of cost-effectiveness evaluations reported inconsistency in the way that ‘benefits and harms’ were conceptualised in NBS models with 20% containing no discussion of this important area.^47^ Another review reported a high level of variation between NBS models in key areas affecting model structure, outcome measures and decision-rules.^48^^,^^49^ Some fundamental challenges were identified by these reviews, such as limited long-term data relating to the modelled comparators in both routine clinical practice and screening programmes.

These limitations in the models create uncertainty about their generalisability from one healthcare jurisdiction to another. This limits the ability of decision-making bodies to adopt or adapt modelling studies developed in other countries. Addressing issues such as those raised in the reviews may, in the long-term, improve the value of a modelling-based approach to the assessment of evidence in NBS.

More generally, the limitations imposed by scarce and/or low-quality data for key model inputs returns to the problem which the use of modelling itself seeks to address. A model can only be as good as the data used to populate it. Therefore, input of good quality data into a model is key and the UKNSC uses pilots and in-service evaluations to generate information required for decision-making (See Boxes 1 and 2).

Box 1 **In-service evaluation: the example of medium chain acyl-CoA dehydrogenase deficiency (MCADD).**

**Aims**

• The aim of the evaluation was to report on:
  o How accurately children with and without MCADD would be identified by NBS.
  o The experiences of families of children diagnosed through screening.
  o The early childhood outcomes for affected children detected by screening.

**Results**

• From March 2004 to February 2008, over 1.5 million babies in England were screened for MCADD using tandem mass spectrometry for quantitation of octanoylcarnitine (C8).^50^
• MCADD was confirmed in 147 of 190 babies with a positive screening result giving an overall positive predictive value (PPV) of 77% (147/190, 95% CI: 71–83%).^50^
• A paediatric surveillance system was used to monitor cases presenting clinically before the day of screening and those missed by the screening test.^50^
• Parents highlighted several issues including: coming to terms with the diagnosis, increased significance of feeding, uncertainty in relation to the baby health status.^51^
• Short-term clinical outcomes in the screened cohort reported: one serious clinical episode, one case of developmental delay and one death.^52^
• Based on the evaluation, the UKNSC had the information needed to recommend the introduction of screening for MCADD.

**Important points to consider**

• In-service evaluation activities are resource intensive and require substantial and careful planning across the wider healthcare system.
• During implementation, the distinction between pilot/in-service evaluation and an established screening programme must be made clear to parents to enable them to make an informed decision about whether to screen their baby.

Box 2 **Combined in-service evaluation and modelling example: severe combined immunodeficiency (SCID).**

• Newborn screening for SCID came to the attention of the UKNSC through an open call for bloodspot topics.
• Stakeholders’ involvement helped to inform the scope of a rapid review of the evidence on screening for SCID.
• A public consultation on the review’s findings took place in 2012 and attracted over 200 responses from national organisations, individual professionals and members of the public.
• Evidence gaps were identified by the review but multiple attempts to stimulate research to address these gaps failed to secure funding.
• This led to the UKNSC commissioning a modelling and cost-effectiveness evaluation of SCID screening which informed the recommendation to fund an in-service evaluation.^53^

**The in-service evaluation**

• Launched in September 2021 in England, covering around two-thirds of the newborn population and expected to initially run for two years.
• The aim is to help assess if screening does more good than harm by determining:
  o How many more babies with SCID are found by screening compared to babies found by cascade testing.
  o How many babies with immune disorders other than SCID would be detected, what benefits they might gain or harms they might suffer.
  o Whether the health of affected babies would be improved.
• Led by a multi-disciplinary group of stakeholders, including PPV representatives and modellers who will use data from the evaluation to update the original cost-effectiveness model and help inform the final UKNSC recommendation on screening for SCID.
• The results of the in-service evaluation will be made available to stakeholders as part of a public consultation process.^54^

Other tools to potentially improve research in NBS, recognised by EURORDIS (principles 10 and 11) are the use of stored dried bloodspots (DBS) and registries. Linked together, DBS, registries, and in-service evaluations may provide the resources to construct case series, case-control and retrospective cohort studies. These could add value to decision-making criteria, in particular those addressing epidemiology, natural history including genotype/phenotype relationship and treatment outcomes and could be used to inform key parameters in modelling studies.

The need to explore the potential of integrating these resources in the UK may be becoming more pressing as the GEL research on the use of whole genome sequencing in newborns, alongside the current NBS programme, gains momentum.

### Stakeholder involvement

The UKNSC and the NHS NBS programme have established mechanisms to involve stakeholder groups at all points of policy development and programme implementation (See Box 2).

However, there is significant divergence between specific EURORDIS recommendations and UK practice, for example, at particular points of the UKNSC decision-making process. While there is PPV membership of UKNSC and its reference groups, disease specific groups and groups of topic area experts are not invited to attend UKNSC meetings and the kind of preferential partnership between these groups which EURORDIS recommend has not been incorporated into UK screening decision-making. The divergence between the UKNSC and EURORDIS is therefore not one of participation in the general sense, but the point at which those professionals and parents/patients associated with specific conditions are invited to participate.

As an independent Scientific Advisory Committee covering a broad range of topic areas and disease types, the UKNSC needs to ensure that its stakeholder engagement work is proportionate, flexible, transparent and evenly applied across many stakeholder groups. Nevertheless, the underlying concern that there should be mechanisms for optimal stakeholder participation is certainly a legitimate one.

A recent review of the Committee’s stakeholder engagement activities reported that modelling studies represented a methodology that can create helpful opportunities for engagement of clinical opinion and PPV experience to participate in the evidence gathering for policy-making.^53^

There is consensus in the wider literature about the added value of involving a range of stakeholders in good modelling practice and health economic research.^55^, ^56^, ^57^ Modelling studies provide an opportunity for stakeholders to be involved in an ongoing discussion about the potential effects of a screening programme and the evidence base relating to it. Because models are used as decision tools they also, potentially, position stakeholders in a process where their input can help to directly shape the outcome. However, the actual mechanisms to productively involve stakeholders in health economic models are not well described. Health economic models are notoriously complex pieces of scientific work, and this can sometimes alienate many stakeholders, including clinical and PPV experts. In the absence of well worked-up approaches, health economists have suggested some tools and methods of participation in modelling from other fields such as resource management and environmental planning. These have included meetings, workshops, brainstorming, SWOT (Strengths, Weaknesses, Opportunities, Threats) analyses, questionnaires, animations, and web applications.^58^ The UKNSC has formed expert groups within which models have been developed and these have been useful in eliciting clinical opinion. However the potential of these exercises to incorporate patient experience has remained elusive. Considering how patient and public experience can be productively incorporated into the broader UKNSC process for evaluating topics particularly in the rare diseases area and/or whether this can be realised with reference to examples outside of modelling in healthcare is something which might be explored in future projects.

## Conclusion

Comparing the EURORDIS principles with UK policy and practice has highlighted that the UK is generally well aligned with many of the recommended principles. Taken as a whole, the principles present both a useful reference point and a challenge for the UKNSC and other decision-making bodies operating in the NBS setting.

Consistency across NBS in Europe is a major theme in the EURORDIS principles. This is a far-reaching aspiration. However, it has a practical driver given that many of the barriers in NBS arise because of the rarity of the conditions. Achieving full alignment with all the principles presents some key challenges, namely how to get a better understanding of the reasons for the variation in policy-making and practice, to what extent consistency is desirable and whether variation should be expected and justified.

Some of these, for example actionability will continue to be debated within, and maybe beyond, the NBS setting^24^ and may never reach consensus. Other challenges, such as PPV participation in NBS systems, may be more tractable and subject to continuous improvement as assessment methods and practices evolve. The EURORDIS emphasis on the importance of NBS research infrastructure closely corresponds with a concern also identified by the UKNSC. Work to address this within the bloodspot task group could provide an example of how such relationships might evolve. Note should also be taken of other principles such as those proposed by Scarpa et al.

To facilitate further reflection, screening bodies in other European countries should compare their policy-making and implementation principles and practices with the EURORDIS principles.

## Contributors

Substantial contributions to manuscript conception and design: SL, FS, DE, JM; drafting the initial draft: SL; revising and commenting on the initial draft: FS; critically revising the manuscript for important intellectual content: SL, FS, DE, JM; final approval of the version of the article to be published: SL, FS, DE, JM. The corresponding author attests that all listed authors meet authorship criteria and that no others meeting the criteria have been omitted.

## Ethical approval

Not required.

## Declaration of interests

All authors have completed the ICMJE disclosure form at https://www.thelancet.com/for-authors/forms?section=icmje-coi and declare: SL and JM are employed by the UK National Screening Committee (UKNSC), hosted at the Department of Health and Social Care (DHSC); FS was employed by the UKNSC until October 2022 and she is currently employed by St George's University of London (SGUL); DE is employed by Great Ormond Street Hospital for Children NHS Foundation Trust (GOSH), he has also honorary contracts with NHS England (NHSE) and DHSC to provide advice on matters relating to newborn screening and he is currently the Chair of the UKNSC Bloodspot Task Group; no other relationships or activities that could appear to have influenced the submitted work. The views expressed are those of the author(s) and not necessarily those of the UKNSC, DHSC, SGUL, NHSE or GOSH.
