# Supplementary material for: Policy-making and implementation for newborn bloodspot screening in Europe: a comparison between EURORDIS principles and UK practice
Source: Lancet Reg Health Eur. 2023 Aug 10;33:100714. doi: 10.1016/j.lanepe.2023.100714 (PMC10636270; doi:10.1016/j.lanepe.2023.100714)
Supplement: Supplementary Tables S1–S3 [file mmc1.docx]

# **Supplementary materials: tables**

[**Supplementary materials: tables** 1](#_Toc135149259)

[**Supplementary Table 1. UK NSC criteria for population screening^1^** 2](#_Toc135149260)

[**Supplementary Table 2. EURORDIS key principles for newborn screening^2^** 3](#_Toc135149261)

[**Supplementary Table 3. Scarpa et al. (2022) ten elements for effective operation of NBS programmes in Europe^3^** 4](#_Toc135149262)

## **Supplementary Table 1. UK NSC criteria for population screening^1^**

| **THE CONDITION** | |
| --- | --- |
| **Criterion 1** | The condition should be an important health problem as judged by its frequency and/or severity. The epidemiology, incidence, prevalence and natural history of the condition should be understood, including development from latent to declared disease and/or there should be robust evidence about the association between the risk or disease marker and serious or treatable disease |
| **Criterion 2** | All the cost-effective primary prevention interventions should have been implemented as far as practicable |
| **Criterion 3** | If the carriers of a mutation are identified as a result of screening the natural history of people with this status should be understood, including the psychological implications |
| **THE TEST** | |
| **Criterion 4** | There should be a simple, safe, precise and validated screening test |
| **Criterion 5** | The distribution of test values in the target population should be known and a suitable cut-off level defined and agreed |
| **Criterion 6** | The test, from sample collection to delivery of results, should be acceptable to the target population |
| **Criterion 7** | There should be an agreed policy on the further diagnostic investigation of individuals with a positive test result and on the choices available to those individuals |
| **Criterion 8** | If the test is for a particular mutation or set of genetic variants the method for their selection and the means through which these will be kept under review in the programme should be clearly set out. |
| **THE INTERVENTION** | |
| **Criterion 9** | There should be an effective intervention for patients identified through screening, with evidence that intervention at a pre-symptomatic phase leads to better outcomes for the screened individual compared with usual care. Evidence relating to wider benefits of screening, for example those relating to family members, should be taken into account where available. However, where there is no prospect of benefit for the individual screened then the screening programme should not be further considered |
| **Criterion 10** | There should be agreed evidence based policies covering which individuals should be offered interventions and the appropriate intervention to be offered |
| **THE SCREENING PROGRAMME** | |
| **Criterion 11** | There should be evidence from high quality randomised controlled trials that the screening programme is effective in reducing mortality or morbidity. Where screening is aimed solely at providing information to allow the person being screened to make an “informed choice” (such as Down’s syndrome or cystic fibrosis carrier screening), there must be evidence from high quality trials that the test accurately measures risk. The information that is provided about the test and its outcome must be of value and readily understood by the individual being screened |
| **Criterion 12** | There should be evidence that the complete screening programme (test, diagnostic procedures, treatment/ intervention) is clinically, socially and ethically acceptable to health professionals and the public |
| **Criterion 13** | The benefit gained by individuals from the screening programme should outweigh any harms, for example from overdiagnosis, overtreatment, false positives, false reassurance, uncertain findings and complications |
| **Criterion 14** | The opportunity cost of the screening programme (including testing, diagnosis and treatment, administration, training and quality assurance) should be economically balanced in relation to expenditure on medical care as a whole (value for money). Assessment against this criteria should have regard to evidence from cost benefit and/or cost effectiveness analyses and have regard to the effective use of available resource |
| **IMPLEMENTATION CRITERIA** | |
| **Criterion 15** | Clinical management of the condition and patient outcomes should be optimised in all health care providers prior to participation in a screening programme |
| **Criterion 16** | All other options for managing the condition should have been considered (such as improving treatment or providing other services), to ensure that no more cost effective intervention could be introduced or current interventions increased within the resources available |
| **Criterion 17** | There should be a plan for managing and monitoring the screening programme and an agreed set of quality assurance standards |
| **Criterion 18** | Adequate staffing and facilities for testing, diagnosis, treatment and programme management should be available prior to the commencement of the screening programme |
| **Criterion 19** | Evidence-based information, explaining the purpose and potential consequences of screening, investigation and preventative intervention or treatment, should be made available to potential participants to assist them in making an informed choice |
| **Criterion 20** | Public pressure for widening the eligibility criteria for reducing the screening interval, and for increasing the sensitivity of the testing process, should be anticipated. Decisions about these parameters should be scientifically justifiable to the public |

## **Supplementary Table 2. EURORDIS key principles for newborn screening^2^**

| **Key Principle 1** | Screening should identify opportunities to help the newborn and the family as broadly as possible. That is, screening should identify actionable diseases including treatable diseases |
| --- | --- |
| **Key Principle 2** | NBS should be organised as a system with clearly defined roles, responsibilities, accountability and communication pathways that are embedded into the national health care system and recognised as a mechanism for earlier diagnosis of actionable conditions as part of the broader care pathway |
| **Key Principle 3** | The family of the newborn who has been diagnosed through NBS should be provided with psychological, social and economic support by the competent national health authorities |
| **Key Principle 4** | All stakeholders should be included in the different stages of the NBS process |
| **Key Principle 5** | Transparent and robust governance for expanding NBS programmes is needed. Every country/region should have a clearly defined transparent, independent, impartial and evidence-based process for deciding which conditions are covered by the NBS programme that includes all stakeholders |
| **Key Principle 6** | Governance of NBS programmes should be explicit, comprehensive, transparent and accountable to national authorities |
| **Key Principle 7** | The evaluation process on the inclusion/exclusion of diseases in NBS programmes needs to be based on the best available evidence, reflecting health economic evidence but not determined only by health economics |
| **Key Principle 8** | Information and education of all stakeholders on rare diseases and the whole NBS process is essential for a broad and fair implementation of NBS programmes |
| **Key Principle 9** | European-wide standards addressing the timing, sample collection methods, follow-up, and information shared with parents are needed to guarantee uniformity and quality throughout the process |
| **Key Principle 10** | Blood spot samples should be stored in national biobanks for quality control and research purposes while ensuring appropriate measures for data access as well as robust safeguards for data protection and privacy are in place |
| **Key Principle 11** | ERN affiliated centres should be integrated in the care pathways of the different Healthcare systems and should be considered as preferential partners in providing recommendations on NBS policies |

## **Supplementary Table 3. Scarpa et al. (2022) ten elements for effective operation of NBS programmes in Europe^3^**

| **Element 1** | Selection of (new) conditions in NBS panels should be based on published criteria, the procedures should be standardised, open to public scrutiny and the result of deliberations should be published |
| --- | --- |
| **Element 2** | Information (preferably communicated during pregnancy) describing the diseases to be tested and the implications of a positive result should be available to parents to permit an informed choice concerning participation |
| **Element 3** | Clear case definitions of the screened disorders should be determined when screening is being planned |
| **Element 4** | Screening should be undertaken in laboratories whose accreditation demonstrates compliance with international standards for laboratory performance (e.g., ISO15189) |
| **Element 5** | Laboratories and programmes should be able to produce data on key performance indicators relating to the entire NBS process, including blood sampling, transport conditions, blood spot quality, time to generate a laboratory result and refer screen positive cases |
| **Element 6** | Information should be available to parents at the time of clinical referral, the first contact should be with an experienced physician able to offer support, and, when appropriate, genetic counselling should be provided |
| **Element 7** | Confirmatory testing should be established and consistently applied with a short and defined turnaround time to allay parental anxiety and stress |
| **Element 8** | Plans to assess long term outcome data should be in place and reported |
| **Element 9** | Screen negative results should be reported to all parents and form part of the child health record |
| **Element 10** | Policies to store and access residual blood-spot samples should be defined and practice monitored. NBS programs should be coordinated, and performance managed on a national basis to encourage continuous improvement |

**References**

1. UK National Screening Committee. Criteria for a population screening programme. Accessed: 24/10/2022; Available from: <https://www.gov.uk/government/publications/evidence-review-criteria-national-screening-programmes/criteria-for-appraising-the-viability-effectiveness-and-appropriateness-of-a-screening-programme>.

2. EURORDIS Rare Diseases Europe. Key principles for newborn screening 2021. Accessed: 05/10/2022; Available from: <https://download2.eurordis.org/documents/pdf/eurordis_nbs_position_paper.pdf>.

3. Scarpa M, Bonham JR, Dionisi-Vici C, Prevot J, Pergent M, Meyts I, et al. Newborn screening as a fully integrated system to stimulate equity in neonatal screening in Europe. *The Lancet Regional Health–Europe* 2022;**13**.
